# Relationship between oxidative balance indicators and Chronic Kidney Disease

**DOI:** 10.1371/journal.pone.0315344

**Published:** 2025-01-03

**Authors:** Xinyun Chen, Fangyu Shi, Wenhui Yu, Chang Gao, Shenju Gou, Ping Fu

**Affiliations:** 1 Department of Health Management, Health Management Center, General Practice Center, West China Hospital, Sichuan University, Chengdu, China; 2 Department of Nephrology, Institute of Kidney Diseases, West China Hospital, Sichuan University, Chengdu, China; National Healthcare Group, SINGAPORE

## Abstract

**Introduction:**

Chronic Kidney Disease (CKD) is a growing global health issue, affecting approximately 9.1% of the world’s population. Oxidative stress is believed to play a key role in CKD development, with indicators such as the Oxidative Balance Score (OBS), Pro-Oxidant-Antioxidant Balance (PAB), and Total Antioxidant Capacity (TAC) being of particular interest. However, their association with CKD remains unclear.

**Methods:**

This study conducted a cross-sectional analysis using data from the National Health and Nutrition Examination Survey (NHANES) 2009–2018. A total of 18,951 participants were included after applying specific inclusion criteria. Logistic regression models and restricted cubic spline regression were employed to examine the associations between these oxidative balance indicators and CKD. Subgroup and interaction analyses were also conducted for further data analyses. Finally, ROC curve analysis was used to assess the predictive performance of these indicators for CKD risk.

**Results:**

After adjusting for various confounding factors, higher levels of OBS and PAB were significantly associated with a reduced risk of CKD (OR = 0.97, 95% CI: 0.96–0.99, *P* < 0.001; OR = 0.94, 95% CI: 0.92–0.97, *P* < 0.001, respectively). The ORs for the highest quartiles of OBS and PAB were 0.60 (95% CI: 0.49–0.75, *P* < 0.001) and 0.77 (95% CI: 0.63–0.94, *P* = 0.013), respectively. In contrast, TAC showed no significant association with CKD. ROC curve analysis further indicated that OBS had a superior predictive ability for CKD risk (AUC = 0.579) compared to PAB (AUC = 0.519) and TAC (AUC = 0.492).

**Conclusion:**

The study suggests that oxidative balance indicators, particularly OBS and PAB, are inversely associated with CKD risk, while TAC showed no significant link. OBS demonstrated the strongest predictive ability among the indicators. These findings highlight the potential role of oxidative balance in CKD prevention. Further research is needed to confirm these associations in diverse populations and to explore the underlying mechanisms.

## 1. Introduction

Chronic Kidney Disease (CKD) is a significant global health issue. Recent statistics indicate that about 9.1% of the world’s population is affected by CKD, totaling an estimated 697.5 million people [1, 2]. Additionally, CKD is responsible for nearly 1.2 million deaths each year, ranking it as the twelfth leading cause of death worldwide [1, 2].

Oxidative balance, referring to the equilibrium between the production of reactive oxygen species (ROS) and the body’s ability to neutralize them through antioxidant defenses, plays a crucial role in the progression of CKD through several biological mechanisms [3]. In CKD, increased ROS can be attributed to multiple factors, including inflammation, uremic toxins, and metabolic disturbances [4]. Elevated ROS levels can cause direct damage to renal cells, leading to structural and functional impairments within the kidney. Furthermore, ROS contributes to the activation of pro-inflammatory pathways and fibrotic processes, exacerbating kidney injury [5]. It also interferes with vascular function, promoting endothelial dysfunction and hypertension, which are key risk factors for CKD progression [6]. Additionally, ROS has been linked to alterations in lipid and protein metabolism, further aggravating kidney damage [7]. Thus, ROS not only accelerates the deterioration of renal function but also increases the risk of cardiovascular complications commonly associated with CKD. A Cochrane review, which included 95 studies with 10,468 participants, found that antioxidants likely reduced the progression to kidney failure, with a risk reduction (RR 0.65), suggesting a potential beneficial effect on kidney function [8]. Understanding the role of ROS in CKD pathogenesis is critical, as it presents a potential target for therapeutic interventions aimed at slowing disease progression and improving patient outcomes.

In this context, our focus has turned to several oxidative balance indicators, namely oxidative balance score (OBS), pro-oxidant-antioxidant balance (PAB) and total antioxidant capacity (TAC). OBS serves as a comprehensive indicator of oxidative stress by integrating various dietary and lifestyle factors that influence the balance between pro-oxidants and antioxidants in the body [9]. In the context of oxidative stress, OBS captures the cumulative impact of behaviors and dietary patterns on the body’s ability to counteract oxidative damage. A higher OBS indicates a greater antioxidant influence, suggesting a stronger capacity to neutralize reactive oxygen species (ROS) and mitigate oxidative damage [10]. Conversely, a lower OBS reflects a pro-oxidant-dominated state, which may lead to increased oxidative stress and a higher risk of oxidative stress-related diseases [11]. The PAB is a measure that reflects the equilibrium between oxidative and antioxidative forces within the body [12]. Given the complexity of directly measuring each individual agent’s pro-oxidant or antioxidant potential in biological fluids (e.g., saliva, urine, serum), researchers developed an indirect method to estimate PAB based on lifestyle, nutritional, and medication factors [12]. This approach allows for a more holistic assessment of an individual’s PAB status, considering the pooled effects of various health behaviors on oxidative stress. TAC is a comprehensive measure of the overall antioxidant defenses in the body, encompassing both enzymatic and non-enzymatic antioxidants [13]. TAC reflects the cumulative action of various antioxidants in neutralizing ROS and free radicals. In the context of oxidative stress, TAC serves as an indicator of the body’s ability to counterbalance the harmful effects of excessive ROS production [13]. A higher TAC value suggests a robust antioxidant defense system, which is capable of mitigating oxidative damage to cellular components, including lipids, proteins, and DNA [14]. Conversely, a lower TAC level may indicate a compromised antioxidant defense, making cells and tissues more vulnerable to oxidative stress and its associated pathological consequences [14].

However, the association between various oxidative balance indicators and CKD is not yet clearly understood. To address this, a cross-sectional study was conducted to examine the correlations of OBS, PAB, and TAC with CKD using data from the National Health and Nutrition Examination Survey (NHANES), and a comparative evaluation of the diagnostic performance of these three indices for CKD was further performed.

## 2. Materials and methods

### 2.1 Study population

NHANES is an ongoing series of cross-sectional surveys designed to assess the health and nutritional status of the U.S. population (https://www.cdc.gov/nchs/nhanes/). The NHANES protocol was approved by the NCHS Research Ethics Review Board, and informed consent was obtained from all participants. This analysis utilized data collected between 2009 and 2018.

Inclusion Criteria:

Participants from the NHANES surveys conducted between 2009 and 2018.

Exclusion Criteria:

1. Participants with missing data on oxidative balance indicators (OBS, PAB, or TAC) components.
2. Participants with incomplete information on age, race, serum creatinine (Scr), or urine albumin-to-creatinine ratio (UACR).
3. Participants under the age of 20 years
4. Participants who were pregnant.
5. Participants with implausible energy intake, defined as <800 kcal/day or >4,200 kcal/day for males and <500 kcal/day or >3,500 kcal/day for females.

**Fig 1:**
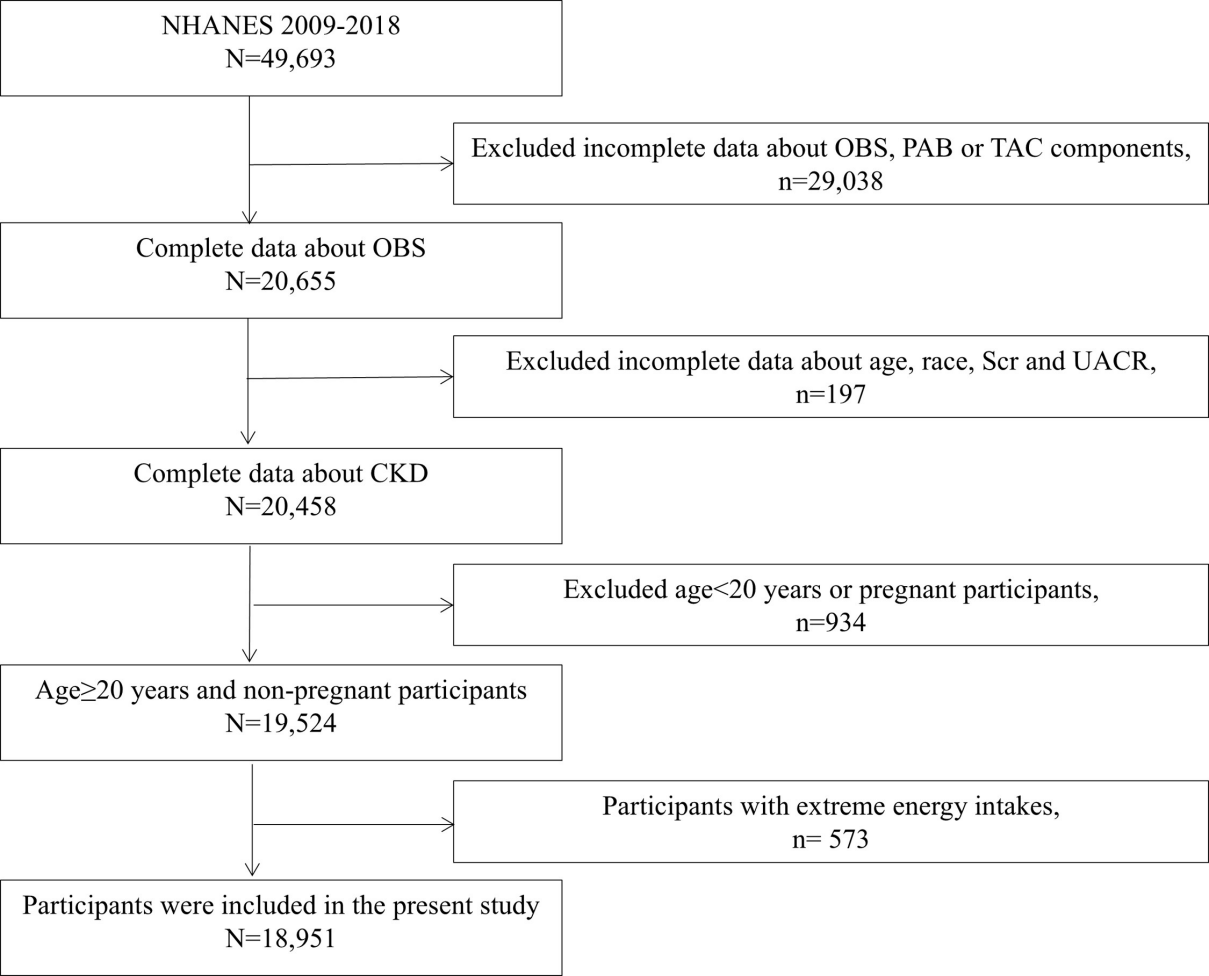
A flowchart illustrating the selection process of study participants.

After applying these criteria, the final study sample consisted of 18,951 participants (Fig 1).

### 2.2 Data collection

#### 2.2.1 Exposure variable

The Oxidative Balance Score (OBS) includes 16 dietary and 4 lifestyle components [11, 15], categorized into 5 pro-oxidants (total fat, iron, alcohol, BMI, cotinine) and 15 antioxidants (e.g., dietary fiber, carotene, vitamins B2, B6, B12, C, E, minerals like calcium, magnesium, zinc, copper, selenium, and physical activity). Except for alcohol, all components were divided into gender-specific tertiles. Antioxidants were scored 0 to 2 (lowest to highest tertile), while pro-oxidants were scored inversely (2 to 0). The total OBS, summing these scores, indicates a more favorable oxidative balance with higher values. S1 Table details the classifications and scores.

The Pro-Oxidant/Antioxidant Balance (PAB) score was calculated following methods outlined in previous studies [12, 16]. In this analysis, PAB components included both pro-oxidant factors (overweight/obesity, smoking, drinking) and antioxidant factors (physical activity, diet quality, fruit and vegetable intake). Each component was categorized into three levels, with pro-oxidant factors assigned scores of 0, -1, or -2, and antioxidant factors assigned scores of 0, +1, or +2. The overall PAB score was derived by summing these components, resulting in a range from -6 to 8, where higher scores indicate a favorable shift towards antioxidant dominance (S2 Table).

To calculate the total antioxidant capacity (TAC), eight antioxidative vitamins were considered: vitamin A, vitamin C, vitamin E, α-carotene, β-carotene, β-cryptoxanthin, lycopene, and lutein-zeaxanthin as previous reported [14]. Floegel et al. developed and validated an algorithm to create a TAC database for the U.S. diet [17]. The TAC level is determined by multiplying the daily intake of each of these eight antioxidants by their respective antioxidant capacities and then summing these values to obtain the overall dietary TAC for each individual. The specific calculation formula is as follows:

$$\text{TAC}=\sum \left[\text{Antioxidant}\;\text{content}\;(\frac{100\;\text{g}}{d})\;\text{x}\;\text{Antioxidant}\;\text{capacity}\;(\frac{\text{mg}\;\text{VCE}}{100\;\text{g}})\right]$$

#### 2.2.2 Outcome variable

CKD is identified by a persistent abnormality in kidney structure or function for at least three months, marked by either a decreased estimated glomerular filtration rate (eGFR) or the presence of albuminuria [18]. A decreased eGFR is defined as a value below 60 mL/min/1.73 m², while albuminuria is indicated by a urine albumin-to-creatinine ratio (UACR) of 30 mg/g or higher [18]. The eGFR was estimated using the Chronic Kidney Disease Epidemiology Collaboration (CKD-EPI) equation, based on serum creatinine (Scr) levels. Urinary albumin concentrations were measured using a solid-phase fluorescence immunoassay, and urinary creatinine levels were assessed with an enzymatic method.

#### 2.2.3 Covariates

Covariate data were obtained through questionnaires, physical measurements, and laboratory tests. The surveys collected details on age, sex, ethnicity, smoking habits, alcohol intake, calorie consumption, and health conditions, including diabetes mellitus, hypertension, cardiovascular diseases, and cancer. Physical assessments provided measurements such as Body Mass Index (BMI). Cardiovascular disease (CVD) was identified in individuals with a history of congestive heart failure, coronary artery disease, angina, or myocardial infarction [19]. Hypertension was defined as having a systolic pressure of ≥130 mmHg, diastolic pressure of ≥90 mmHg, a previous diagnosis of high blood pressure, or the use of blood pressure-lowering medication [20]. Diabetes mellitus (DM) was determined based on a prior diagnosis, use of diabetes medication or insulin, fasting blood sugar ≥126 mg/dL, post-oral glucose intake levels ≥200 mg/dL, or HbA1c ≥6.5% [21]. Household income was grouped according to the Federal Poverty Level (FPL) into three categories: ≤130% (reference), >130–350%, and >350% [22]. Alcohol consumption was classified into never (fewer than 12 drinks in a lifetime), low-to-moderate (up to one drink daily for women and two for men in the past year), and high (more than one drink daily for women and two for men in the past year). Smoking status was categorized as never (fewer than 100 cigarettes in a lifetime), former (over 100 cigarettes but not currently smoking), and current smokers (over 100 cigarettes and currently smoking). Average daily calorie intake was assessed over two days using dietary data.

### 2.3 Statistical analyses

Statistical analyses were performed following the guidelines of the Centers for Disease Control, utilizing a weighted statistical approach. Continuous variables were presented as adjusted means ± standard deviations or as medians with interquartile ranges, while categorical variables were reported as unweighted counts with their corresponding weighted percentages. Four logistic regression models were constructed: Model 1 had no adjustments for confounders, Model 2 included adjustments for demographic factors such as age, sex, and ethnicity, Model 3 further adjusted for lifestyle factors like alcohol consumption, smoking status, BMI, physical activity, and dietary caloric intake, and Model 4 additionally accounted for health history, including cardiovascular disease, hypertension, and diabetes mellitus (DM).

To assess dose-response relationships between oxidative balance indicators and CKD, a restricted cubic spline (RCS) regression with four knots at the 5th, 35th, 65th, and 95th percentiles of the oxidative balance indicator values was used. Subgroup and interaction analyses were conducted to explore potential interactive effects among various covariates. The discriminatory capacity of each oxidative balance indicator for identifying CKD was evaluated using ROC curve analysis. All statistical analyses were performed using R software, version 4.2.2 (https://www.R-project.org; R Foundation, Austria), with statistical significance set at *P* < 0.05.

## 3. Results

### 3.1 Characteristics of the participants

Table 1 summarizes the baseline characteristics of participants categorized based on the presence of CKD. Among the 18,951 participants, 15,729 were classified as non-CKD, and 3,222 as CKD. The CKD group exhibits a higher mean age, a lower proportion of males, and an increased prevalence of conditions such as hypertension, diabetes, and cardiovascular disease. Additionally, notable differences are observed in lifestyle factors, including smoking and alcohol consumption, as well as clinical parameters such as BMI, hemoglobin, and serum creatinine, highlighting potential risk factors or consequences associated with CKD.

**Table 1:** Baseline characteristics of participants classified by Chronic Kidney Disease (CKD), weighted.

Characteristics^†^	Overall (*n* = 18,951)	Non-CKD (*n* = 15,729)	CKD (*n* = 3,222)	*P*-value
Male, *n* (%)	9,135 (48.4)	7,653 (49.3)	1,482 (42.5)	<0.001
Age, years	47.67 (16.92)	45.53 (15.93)	60.93 (16.84)	<0.001
Race, *n* (%)				
Mexican American	2,760 (8.3)	2,367 (8.5)	393 (6.6)	
Other Hispanic	1,995 (5.9)	1,706 (6.1)	289 (4.8)	
Non-Hispanic white	7,824 (67.8)	6,299 (67.3)	1,525 (70.9)	<0.001
Non-Hispanic Black	3,857 (10.1)	3,151 (9.9)	706 (11.4)	
Others	2,515 (7.9)	2,206 (8.2)	309 (6.3)	
Education level, *n* (%)				
Less than high school	1,646 (4.3)	1,254 (3.9)	392 (6.7)	
High school	6,521 (31.2)	5,297 (30.4)	1,224 (35.9)	<0.001
More than high school	10,784 (64.5)	9,178 (65.7)	1,606 (57.4)	
Household income, *n* (%)				
0–130% FPL	5,753 (20.3)	4,723 (20.0)	1,030 (22.5)	
>130–350% FPL	7,169 (35.5)	5,788 (34.4)	1,381 (41.9)	<0.001
>350% FPL	6,029 (44.2)	5,218 (45.6)	811 (35.6)	
Smoking, *n* (%)				
No	11,100 (58.1)	9,341 (58.8)	1,759 (54.1)	
Former	4,299 (24.2)	3,336 (22.9)	963 (31.9)	<0.001
Current	3,552 (17.7)	3,052 (18.3)	500 (14.0)	
Drinking, *n* (%)				
No	5,863 (24.0)	4,521 (22.1)	1,342 (35.5)	
Low-to-moderate	11,643 (66.9)	9,993 (68.7)	1,650 (55.6)	<0.001
Heavy	1,445 (9.1)	1,215 (9.1)	230 (8.9)	
Marital status, *n* (%)				
Married	9,741 (55.8)	8,131 (56.0)	1,610 (54.1)	<0.001
Widowed	1,369 (5.4)	802 (3.8)	567 (15.3)	
Divorced	2,064 (10.1)	1,641 (9.7)	423 (12.5)	
Separated	606 (2.2)	498 (2.2)	108 (2.4)	
Never married	3,592 (18.3)	3,235 (19.6)	357 (10.1)	
Living with partner	1,579 (8.3)	1,422 (8.7)	157 (5.4)	
Hypertension, *n* (%)	6,792 (31.9)	4,757 (27.4)	2,035 (59.7)	<0.001
Diabetes, *n* (%)	3,494 (14.0)	2,193 (10.7)	1,301 (34.7)	<0.001
CVD, *n* (%)	1,447 (6.3)	787 (4.5)	660 (17.9)	<0.001
Cancer, *n* (%)	1,812 (10.5)	1,217 (9.0)	595 (20.1)	<0.001
BMI, kg/m^2^	29.15 (6.86)	28.95 (6.74)	30.40 (7.40)	<0.001
MET, *n* (%)				
<600 min/week	7,200 (33.4)	5,493 (31.0)	1,707 (48.4)	
600–3999 min/week	6,944 (39.6)	5,972 (40.6)	972 (33.3)	<0.001
≥4000 min/week	4,807 (27.0)	4,264 (28.4)	543 (18.4)	
Calorie, kcal/day	1955.5 (1523.50, 2465.00)	1981.50 (1550.50, 2494.48)	1774.00 (1382.00, 2242.33)	<0.001
Hemoglobin, g/L	14.21 (1.43)	14.27 (1.39)	13.79 (1.62)	<0.001
Albumin, g/L	42.71 (3.34)	42.89 (3.30)	41.57 (3.40)	<0.001
TC, mmol/L	4.91 (4.24, 5.64)	4.91 (4.24, 5.64)	4.84 (4.16, 5.69)	0.274
TG, mmol/L	1.11 (0.76, 1.64)	1.07 (0.74, 1.60)	1.25 [0.88, 1.82]	<0.001
HDL, mmol/L	1.40 (0.43)	1.40 (0.42)	1.38 (0.48)	0.132
LDL, mmol/L	2.95 (0.89)	2.96 (0.88)	2.88 (0.98)	0.002
HbA1c, %	5.63 (0.90)	5.54 (0.75)	6.16 (1.40)	<0.001
Serum creatinine, μmol/L	75.14 (63.65, 87.52)	74.26 (62.76, 84.86)	89.28 (70.72, 111.38)	<0.001
Urea nitrogen, mmol/L	4.64 (3.57, 5.71)	4.64 (3.57, 5.71)	5.71 (4.28, 7.85)	<0.001
eGFR,ml/min/1.73m^2^	94.03 (21.76)	97.53 (18.10)	72.41 (28.86)	<0.001
UACR, mg/g	6.58 (4.40, 11.56)	6.03 (4.18, 9.26)	41.10 (12.15, 92.83)	<0.001
Decreased eGFR, *n* (%)	1,577 (6.8)	-	1,577 (48.6)	<0.001
Proteinuria, *n* (%)	2,179 (9.1)	-	2,179 (65.2)	<0.001

FPL, family income to poverty; CVD, cardiovascular disease; BMI, body mass index; MET, metabolic equivalent; TC, total cholesterol; TG, triglyceride; eGFR, Estimated glomerular filtration rate; UACR, urine albumin/creatinine ratio; CKD, chronic kidney disease.

* *P* <0.05

^**†**^ *n* (%): Unweighted numbers (weighted percentage).

### 3.2 Association between oxidative balance indicators and kidney function

Table 2 displays the logistic regression analysis assessing the associations between oxidative balance indicators (OBS, PAB, and TAC) and CKD across four models. In Model 4, a continuous increase in OBS is linked to a lower CKD risk, with an odds ratio (OR) of 0.97 (95% CI: 0.96, 0.99; *P* < 0.001). When OBS is stratified into quartiles, participants in higher quartiles (Q2, Q3, and Q4) demonstrate a significantly reduced risk of CKD relative to the reference group (Q1). The ORs for these quartiles are 0.83 (95% CI: 0.70, 0.99; *P* = 0.037), 0.68 (95% CI: 0.57, 0.81; *P* < 0.001), and 0.60 (95% CI: 0.49, 0.75; *P* < 0.001), respectively, indicating a clear trend (*P* for trend < 0.001). For PAB in Model 4, the continuous measurement shows an inverse association with CKD, resulting in an OR of 0.94 (95% CI: 0.92, 0.97; *P* < 0.001). In the quartile analysis, only the highest quartile (Q4) presents a lower risk, with an OR of 0.74 (95% CI: 0.62, 0.88; *P* = 0.001). On the other hand, TAC does not exhibit statistically significant associations in Model 4, suggesting no direct relationship with CKD in this context.

**Table 2:** Logistic regression analysis for the associations between oxidative balance indicators and Chronic Kidney Disease, weighted.

	Model 1		Model 2		Model 3		Model 4	
	*OR* (95% CI)	*P*-value	*OR* (95% CI)	*P*-value	*OR* (95% CI)	*P*-value	*OR* (95% CI)	*P* -value
**OBS**								
Continuous	0.97 (0.96, 0.98)	<0.001*	0.97 (0.96, 0.98)	<0.001*	0.97 (0.96, 0.98)	<0.001*	0.97 (0.96, 0.99)	<0.001*
Q1	References		References		References		References	
Q2	0.83 (0.71, 0.96)	0.015	0.79 (0.67, 0.93)	0.004	0.82 (0.70, 0.97)	0.020	0.83 (0.70, 0.99)	0.037
Q3	0.64 (0.56, 0.73)	<0.001*	0.62 (0.54, 0.72)	<0.001*	0.66 (0.56, 0.79)	<0.001*	0.68 (0.57, 0.81)	<0.001*
Q4	0.53 (0.45, 0.62)	<0.001*	0.52 (0.44, 0.62)	<0.001*	0.58 (0.48, 0.71)	<0.001*	0.60 (0.49, 0.75)	<0.001*
*P* for trend		<0.001*		<0.001*		<0.001*		<0.001*
**PAB**								
Continuous	0.99 (0.98, 1.02)	0.915	0.96 (0.93, 0.98)	<0.001*	0.94 (0.91, 0.97)	<0.001*	0.94 (0.92, 0.97)	<0.001*
Q1	References		References		References		References	
Q2	1.05 (0.90, 1.22)	0.554	0.93 (0.78, 1.10	0.387	0.89 (0.74, 1.08)	0.238	0.89 (0.73, 1.08)	0.236
Q3	1.04 (0.91, 1.18)	0.565	0.88 (0.77, 1.02)	0.092	0.85 (0.74, 0.97)	0.020*	0.86 (0.74, 0.99)	0.034*
Q4	0.95 (0.82, 1.11)	0.518	0.75 (0.64, 0.87)	<0.001*	0.72 (0.60, 0.85)	<0.001*	0.74 (0.62, 0.88)	0.001*
*P* for trend		0.762		0.001*		<0.001*		<0.001*
**TAC**								
Continuous	0.97 (0.93, 1.02)	0.289	0.91 (0.86, 0.95)	<0.001*	0.97 (0.91, 1.03)	0.274	0.98 (0.92, 1.04)	0.448
Q1	References		References		References		References	
Q2	1.10 (0.95, 1.26)	0.193	0.99 (0.84, 1.16)	0.874	1.06 (0.90, 1.24)	0.481	1.05 (0.89, 1.23)	0.566
Q3	1.08 (0.95, 1.23)	0.252	0.91 (0.78, 1.05)	0.197	1.02 (0.88, 1.18)	0.805	1.02 (0.88, 1.19)	0.788
Q4	0.97 (0.86, 1.08)	0.568	0.82 (0.72, 0.93)	0.003	0.97 (0.85, 1.10)	0.622	0.99 (0.87, 1.14)	0.954
*P* for trend		0.540		<0.001*		0.499		0.848

* *P* <0.05; OR: weighted odds ratio. Model 1 was a crude model. Model 2 adjusted for age + sex + race, Model 3 was adjusted for Model 1 + BMI + smoking status + drinking status + Physical activity + Calorie intake, and Model 4 was adjusted for Model 2 + CVD + hypertension + diabetes.

Table 3 examines the relationship between oxidative balance indicators and impaired kidney function (eGFR < 60 ml/min/1.73 m²). In Model 4, an increase in OBS is significantly associated with a reduced risk of impaired kidney function, reflected in an OR of 0.96 (95% CI: 0.95, 0.98; P < 0.001). When OBS is divided into quartiles, participants in the higher quartiles (Q3 and Q4) show a notably lower risk than the reference group (Q1). The ORs for Q3 and Q4 are 0.72 (95% CI: 0.56, 0.92; P = 0.011) and 0.51 (95% CI: 0.37, 0.69; *P* < 0.001), respectively, highlighting a significant trend (*P* for trend < 0.001). Regarding PAB in Model 4, the continuous variable indicates an inverse association with impaired kidney function, with an OR of 0.91 (95% CI: 0.87, 0.96; *P* < 0.001). The quartile analysis supports this finding, showing that participants in the highest quartile (Q4) possess a significantly reduced risk of impaired kidney function, with an OR of 0.62 (95% CI: 0.44, 0.86; *P* = 0.005). TAC remains unrelated to impaired kidney function in Model 4, as neither the continuous measure nor the quartile analysis provides significant results.

**Table 3:** Logistic regression analysis for the associations between oxidative balance indicators and impaired kidney function (eGFR<60ml/min/1.73 m^2^), weighted.

	Model 1		Model 2		Model 3		Model 4	
	*OR* (95% CI)	*P*-value	*OR* (95% CI)	*P*-value	*OR* (95% CI)	*P*-value	*OR* (95% CI)	*P*-value
**OBS**								
Continuous	0.96 (0.95, 0.97)	<0.001*	0.96 (0.95, 0.97)	<0.001*	0.97 (0.95, 0.98)	<0.001*	0.96 (0.95, 0.98)	<0.001*
Q1	References		References		References		References	
Q2	0.94 (0.77, 1.15)	0.544	0.88 (0.69, 0.90)	0.261	0.90 (0.71, 1.15)	0.397	0.91 (0.71, 1.18)	0.478
Q3	0.70 (0.57, 0.85)	<0.001*	0.68 (0.55, 0.84)	<0.001*	0.71 (0.55, 0.91)	0.007	0.72 (0.56, 0.92)	0.011
Q4	0.48 (0.38, 0.61)	<0.001*	0.45 (0.35, 0.58)	<0.001*	0.50 (0.36, 0.67)	<0.001*	0.51 (0.37, 0.69)	<0.001*
*P* for trend		<0.001*		<0.001*		<0.001*		<0.001*
**PAB**								
Continuous	1.02 (0.98, 1.06)	0.353	0.95 (0.91, 0.99)	0.031	0.91 (0.86, 0.96)	0.001*	0.91 (0.87, 0.96)	<0.001*
Q1	References		References		References		References	
Q2	1.14 (0.91, 1.42)	0.256	0.91 (0.71, 1.17)	0.473	0.82 (0.63, 1.08)	0.152	0.82 (0.63, 1.06)	0.136
Q3	1.21 (1.02, 1.44)	0.032*	0.93 (0.77, 1.11)	0.407	0.82 (0.68, 0.98)	0.029*	0.82 (0.68, 0.98)	0.028*
Q4	1.02 (0.79, 1.33)	0.860	0.71 (0.77, 0.96)	0.029*	0.61 (0.44, 0.86)	0.005*	0.62 (0.44, 0.86)	0.005*
*P* for trend		0.430		0.044*		0.004*		0.005*
**TAC**								
Continuous	0.92 (0.87, 0.98)	0.009	0.79 (0.73, 0.85)	<0.001*	0.84 (0.78, 0.90)	<0.001*	0.84 (0.78, 0.91)	<0.001*
Q1	References		References		References		References	
Q2	1.23 (1.01, 1.48)	0.039	1.04 (0.83, 1.29)	0.753	1.10 (0.89, 1.37)	0.383	1.09 (0.88, 1.36)	0.434
Q3	1.07 (0.88, 1.29)	0.505	0.78 (0.63, 0.97)	0.028	0.87 (0.71, 1.08)	0.205	0.87 (0.71, 1.08)	0.203
Q4	0.82 (0.69, 0.98)	0.026	0.58 (0.47, 0.72)	<0.001*	0.69 (0.56, 0.83)	<0.001*	0.69 (0.56, 0.84)	<0.001*
*P* for trend		0.012		<0.001*		<0.001*		<0.001*

* *P* <0.05; OR: weighted odds ratio. Model 1 was a crude model. Model 2 adjusted for age + sex + race, Model 3 was adjusted for Model 1 + BMI + smoking status + drinking status + Physical activity + Calorie intake, and Model 4 was adjusted for Model 2 + CVD + hypertension + diabetes.

Table 4 explores the association between oxidative balance indicators and the occurrence of proteinuria (UACR ≥ 30 mg/g). In Model 4, OBS is significantly correlated with a lower risk of proteinuria, where the continuous measure of OBS has an OR of 0.98 (95% CI: 0.97, 0.99, *P* = 0.015). Quartile analysis reveals that participants in the higher quartiles (Q2, Q3, and Q4) exhibit a reduced risk of proteinuria compared to the reference group (Q1). The ORs for Q2, Q3, and Q4 are 0.80 (95% CI: 0.67, 0.95, *P* = 0.013), 0.69 (95% CI: 0.55, 0.87, *P* = 0.002), and 0.71 (95% CI: 0.55, 0.90, *P* = 0.006), respectively, indicating a significant trend (*P* for trend = 0.004). Similarly, the PAB continuous measure in Model 4 also demonstrates a significant inverse relationship with proteinuria, yielding an OR of 0.95 (95% CI: 0.92, 0.99, *P* = 0.009). The quartile analysis further corroborates this inverse association, where the highest PAB quartile (Q4) is linked to a lower risk of proteinuria (OR = 0.77, 95% CI: 0.63, 0.94, *P* = 0.013). Conversely, TAC shows no significant association with proteinuria in Model 4; both the continuous and quartile analyses are non-significant.

**Table 4:** Logistic regression analysis for the associations between oxidative balance indicators and proteinuria (UACR ≥30mg/g), weighted.

	Model 1		Model 2		Model 3		Model 4	
	*OR* (95% CI)	*P*-value	*OR* (95% CI)	*P*-value	*OR* (95% CI)	*P*-value	*OR* (95% CI)	*P*-value
**OBS**								
Continuous	0.96 (0.95, 0.98)	<0.001*	0.97 (0.96, 0.98)	<0.001*	0.98 (0.97, 0.99)	0.004	0.98 (0.97, 0.99)	0.015
Q1	References		References		References		References	
Q2	0.75 (0.63, 0.88)	<0.001*	0.75 (0.63, 0.88)	<0.001*	0.79 (0.66, 0.93)	0.006	0.80 (0.67, 0.95)	0.013
Q3	0.60 (0.51, 0.71)	<0.001*	0.62 (0.52, 0.74)	<0.001*	0.68 (0.55, 0.85)	<0.001*	0.69 (0.55, 0.87)	0.002
Q4	0.54 (0.45, 0.66)	<0.001*	0.57 (0.47, 0.69)	<0.001*	0.67 (0.53, 0.85)	0.001	0.71 (0.55, 0.90)	0.006
*P* for trend		<0.001*		<0.001*		<0.001*		0.004
**PAB**								
Continuous	0.97 (0.94, 1.01)	0.083	0.95 (0.92, 0.98)	<0.001*	0.95 (0.92, 0.98)	0.003*	0.95 (0.92, 0.99)	0.009*
Q1	References		References		References		References	
Q2	0.98 (0.82, 1.16)	0.792	0.92 (0.77, 1.10)	0.358	0.92 (0.76, 1.12)	0.406	0.92 (0.76, 1.13)	0.424
Q3	0.88 (0.74, 1.04)	0.134	0.80 (0.67, 0.96)	0.014*	0.81 (0.68, 0.96)	0.016*	0.81 (0.68, 0.97)	0.025*
Q4	0.84 (0.69, 1.01)	0.064	0.72 (0.60, 0.87)	<0.001*	0.75 (0.61, 0.91)	0.005*	0.77 (0.63, 0.94)	0.013*
*P* for trend		0.042*		<0.001*		0.002*		0.006*
**TAC**								
Continuous	0.99 (0.93, 1.05)	0.630	0.94 (0.89, 1.01)	0.057	1.01 (0.95, 1.08)	0.711	1.03 (0.96, 1.09)	0.437
Q1	References		References		References		References	
Q2	1.07 (0.93, 1.23)	0.332	1.01 (0.87, 1.16)	0.947	1.08 (0.94, 1.25)	0.257	1.07 (0.93, 1.23)	0.364
Q3	1.04 (0.89, 1.23)	0.601	0.94 (0.79, 1.11)	0.437	1.06 (0.89, 1.25)	0.530	1.06 (0.88, 1.26)	0.545
Q4	1.02 (0.88, 1.18)	0.818	0.92 (0.79, 1.06)	0.252	1.09 (0.93, 1.27)	0.271	1.13 (0.97, 1.32)	0.115
*P* for trend		0.906		0.154		0.366		0.156

* *P* <0.05; OR: weighted odds ratio. Model 1 was a crude model. Model 2 adjusted for age + sex + race, Model 3 was adjusted for Model 1 + BMI + smoking status + drinking status + Physical activity + Calorie intake, and Model 4 was adjusted for Model 2 + CVD + hypertension + diabetes.

### 3.3 Restricted cubic spline regression analysis

**Fig 2:**
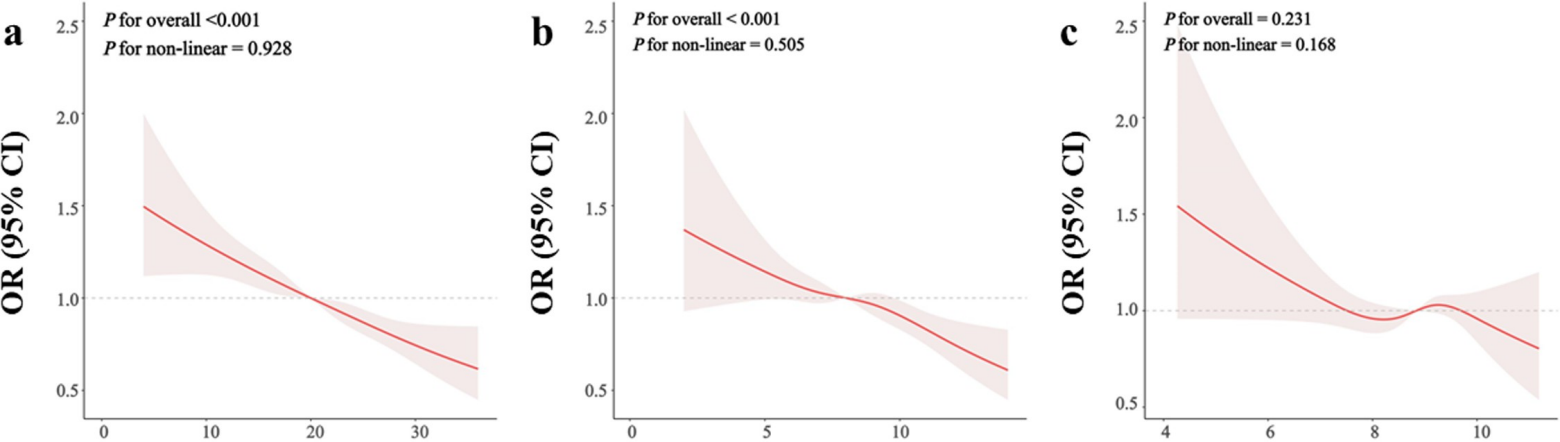
Dose–response relationship between oxidative balance indicators and Chronic Kidney Disease. The association between OBS (a), PAB (b), and TAC (c) with CKD prevalence was assessed using restricted cubic spline regression, adjusting for covariates as in Model 4. The red line indicates the odds ratio, while the pink shading represents the 95% confidence interval.

Fig 2 depicts the dose-response relationships between oxidative balance indicators (OBS, PAB, and TAC) and CKD prevalence using RCS regression, adjusted for covariates. Both OBS and PAB show a clear inverse association with CKD prevalence, as evidenced by the downward slope of the odds ratio (OR) lines and a significant overall effect (*P* for overall < 0.001 for both). Furthermore, the relationships between OBS and PAB with CKD prevalence appear to be linear (*P* for non-linear = 0.928 and 0.505, respectively). In contrast, TAC does not display a significant association with CKD, with both the overall and non-linear tests yielding non-significant results, indicating no clear dose–response relationship. S1 Fig presents the results of the restricted cubic spline regression analysis for the associations between oxidative balance indicators and impaired kidney function (eGFR < 60 ml/min/1.73 m²) as well as proteinuria (UACR ≥ 30 mg/g).

### 3.4 Subgroup analysis

**Fig 3:**
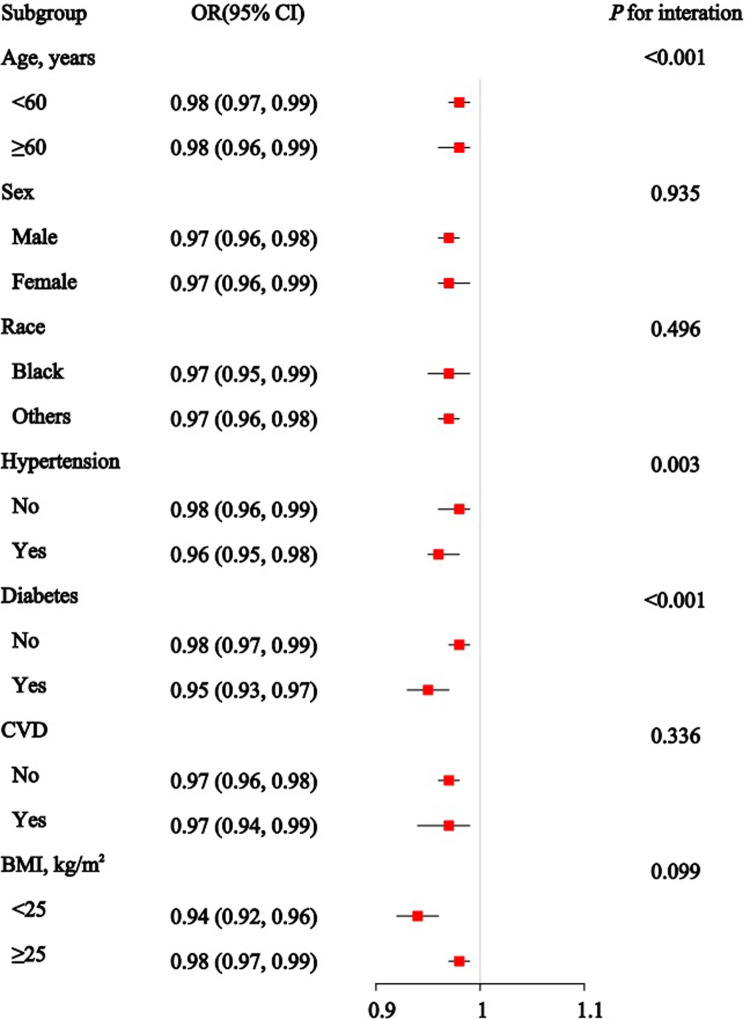
Association between the oxidative balance score (OBS) and Chronic Kidney Disease in subgroup and interactive analyses.

**Fig 4:**
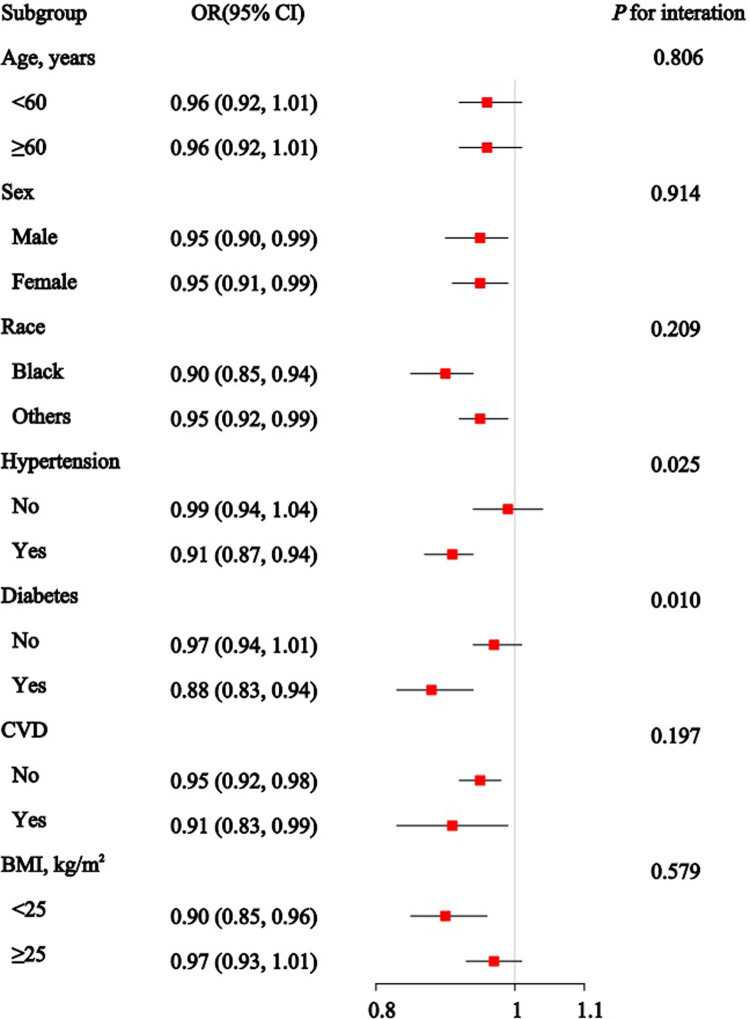
Association between the pro-oxidant/anti-oxidant balance (PAB) and Chronic Kidney Disease in subgroup and interactive analyses.

**Fig 5:**
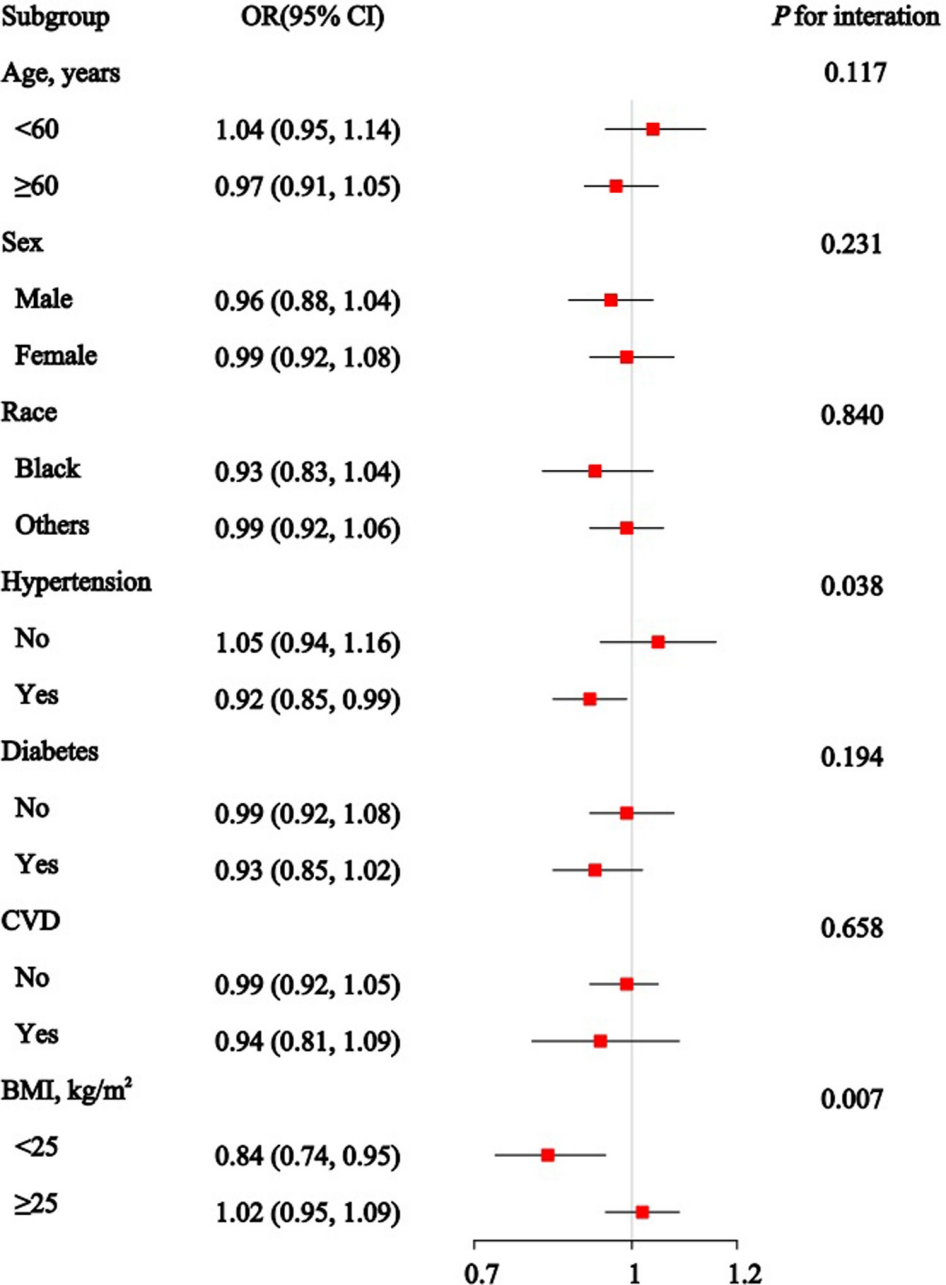
Association between the total antioxidant capacity (TAC) and Chronic Kidney Disease in subgroup and interactive analyses.

Fig 3 presents the associations between the OBS and CKD across various subgroups. The ORs and 95% CIs show an overall inverse relationship between OBS and CKD in all subgroups. Significant interaction effects are observed for age (*P* for interaction < 0.001), hypertension (*P* for interaction = 0.003), and diabetes status (*P* for interaction < 0.001), suggesting that the impact of OBS on CKD risk may vary based on these characteristics. Fig 4 shows a significant reduction in CKD risk with PAB across most subgroups, except for all age groups, individuals without hypertension or diabetes, and those with a BMI of 25 or higher. Significant interaction effects are found for subgroups such as hypertension (*P* for interaction = 0.025) and diabetes status (*P* for interaction = 0.010). Consistent with previous findings, no statistically significant association between TAC and CKD is observed in the subgroup analysis (Fig 5). However, significant interaction effects are noted for hypertension (*P* for interaction = 0.038) and BMI (*P* for interaction = 0.007).

Due to the significant interaction effect of age on the OBS-CKD association, we performed separate RCS regression analyses for individuals aged <60 years and ≥60 years (S2 Fig). Among individuals under 60 years, the non-linearity p-value of 0.766 suggests a predominantly linear association. Similarly, for those aged 60 years or older, the non-linearity p-value of 0.877 indicates no significant evidence of a non-linear trend, further supporting a linear association. In summary, these findings reveal a consistent inverse relationship between OBS and CKD risk across both age groups, with the association being primarily linear.

### 3.5 ROC analyses of oxidative balance indicators in relation to CKD risk

**Fig 6:**
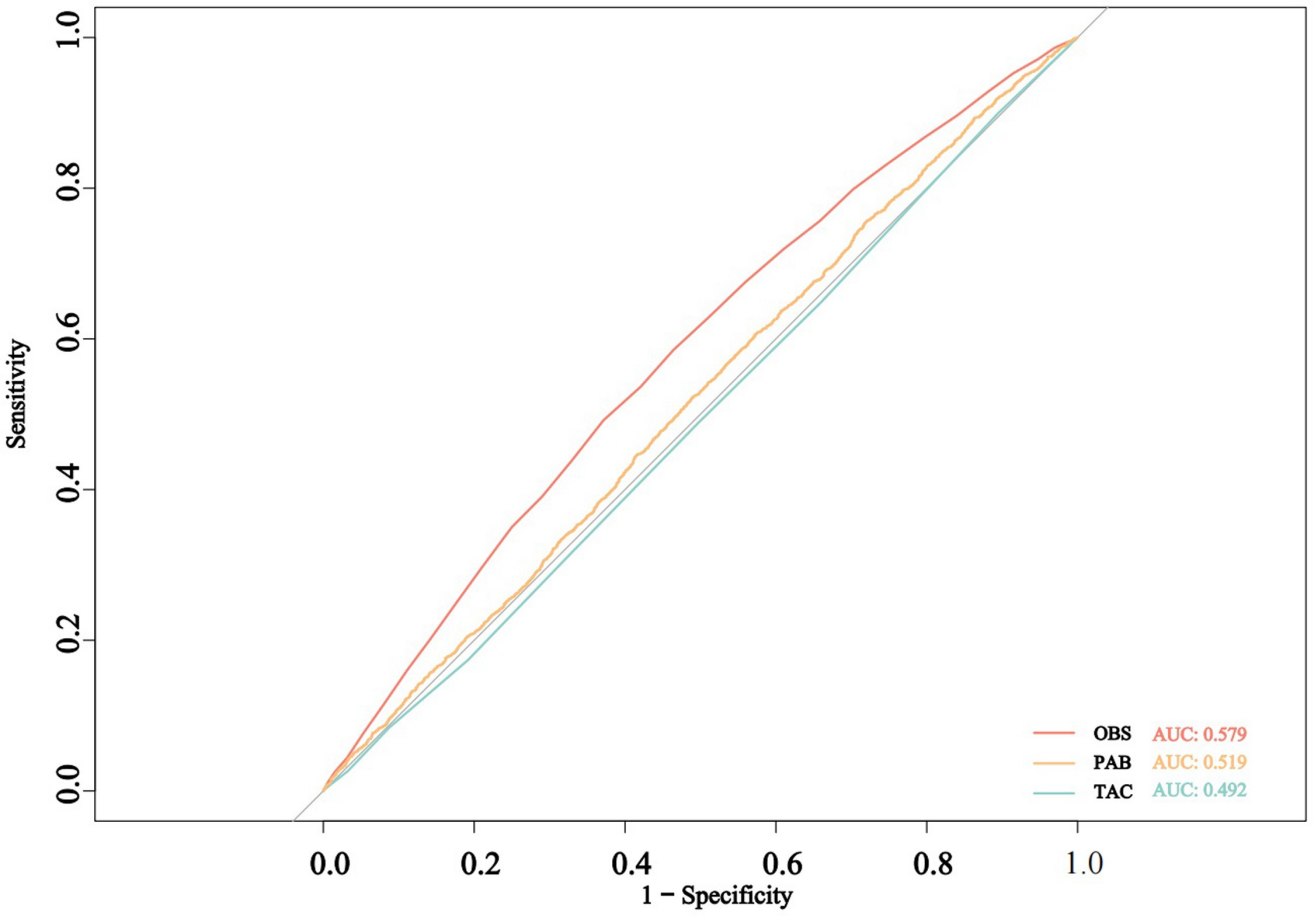
ROC curves for the prediction of CKD by oxidative balance indicators, weighted.

Fig 6 presents the ROC curve comparing the performance of three oxidative balance indicators in predicting CKD risk. The area under the curve (AUC) values are relatively low, with OBS showing an AUC of 0.579, PAB an AUC of 0.519, and TAC an AUC of 0.492. These findings suggest that both PAB and TAC have limited predictive ability, while OBS demonstrates better performance.

## 4. Discussion

This study evaluated the associations of three oxidative balance indicators—OBS, PAB, and TAC—with the risk of CKD. Our findings suggest that while both OBS and PAB are inversely associated with CKD risk, TAC does not show a significant relationship. These results have important implications for understanding the role of oxidative stress in CKD and for selecting appropriate measures to assess oxidative balance.

OBS was identified as the most robust predictor of CKD among the three indicators. Participants in the higher quartiles of OBS exhibited a significantly reduced risk of CKD, impaired kidney function (eGFR < 60 ml/min/1.73 m²), and proteinuria (UACR ≥ 30 mg/g). These results are consistent with previous studies demonstrating the protective effects of OBS [23, 24]. High OBS has been negatively associated with diabetes risk [25], showed a strong inverse correlation with SUA levels and the incidence of hyperuricemia [26], and was linked to a decreased risk of metabolic syndrome (MetS) [27]. By considering both pro-oxidant and antioxidant exposures, OBS provides a comprehensive perspective, potentially explaining its stronger association with CKD risk.

Several studies have provided direct evidence that the OBS is related to oxidative stress levels in the body. Research indicates that a higher OBS, reflecting a more favorable balance between antioxidants and pro-oxidants, is associated with reduced biomarkers of oxidative stress. For example, previous studies have found that individuals with higher OBS scores exhibit lower levels of F2-isoprostanes, a well-established marker of lipid peroxidation and oxidative stress [28, 29]. Furthermore, other research has demonstrated that OBS is inversely related to DNA damage markers, such as 8-hydroxy-2’-deoxyguanosine (8-OHdG), further suggesting that a higher OBS may mitigate oxidative damage at the cellular level [30].

PAB, although also inversely associated with CKD, showed a less robust relationship compared to OBS. This finding is consistent with the literature, which suggests that maintaining a balance between oxidative and antioxidative forces is critical for preventing oxidative stress-related diseases [31]. The harmful impact of hypertension on stroke recurrence (SR) could be alleviated by PAB [12]. However, the effect size for PAB was smaller than that for OBS, which may be due to PAB’s limited scope, as it does not account for the cumulative effect of multiple dietary and lifestyle factors in the same integrative manner as OBS.

In our study, TAC did not show a significant association with CKD, either in continuous or quartile analyses. Despite TAC being widely recognized as a general measure of antioxidant capacity, and numerous studies highlighting its role in various conditions such as stroke [14], cognitive function [32], and depression [33], our findings do not support a significant link between TAC and CKD risk. One possible explanation for this is that TAC reflects the cumulative antioxidant activity in plasma, but it does not capture the dynamic interplay between oxidative stress and antioxidant defenses within specific tissues or microenvironments, such as the kidneys [34]. Additionally, TAC may fail to account for the effects of individual antioxidants that are particularly relevant to kidney pathophysiology. Thus, while TAC provides valuable insight into overall antioxidant status, it may not be sufficiently sensitive to detect the specific oxidative mechanisms involved in CKD development.

When comparing these three indicators, OBS emerges as the most comprehensive and relevant measure of oxidative balance in the context of CKD risk. By incorporating both pro-oxidant and antioxidant components from diet and lifestyle factors, OBS provides a more holistic view of oxidative stress than PAB or TAC alone. PAB offers a direct assessment of the balance between oxidative and antioxidative forces but does not capture the full spectrum of lifestyle influences encompassed by OBS. TAC, although useful in estimating the total antioxidant defenses, falls short in accounting for pro-oxidant exposures, which limits its effectiveness as a predictor of CKD.

Overall, our findings emphasize the complexity of oxidative balance and the importance of using comprehensive measures like OBS to assess its role in CKD risk. Future research should focus on further validating these associations in different populations and exploring the underlying mechanisms through which oxidative balance impacts CKD progression. Additionally, interventions aimed at improving dietary and lifestyle factors, as reflected in the OBS, may provide a promising strategy for CKD prevention.

This study has several limitations. First, the cross-sectional design prevents us from establishing a causal relationship between oxidative balance and CKD. The temporal nature of the associations observed in this study is unclear, and longitudinal research is required to confirm these findings. Second, the reliance on self-reported dietary and lifestyle data to calculate OBS and PAB may introduce reporting bias, potentially affecting the accuracy of these indicators. Additionally, while the study incorporated a comprehensive set of dietary and lifestyle factors in the calculation of OBS and PAB, it did not consider other potential contributors to oxidative stress. These include environmental exposures and genetic predisposition. Lastly, the relatively low area under the curve (AUC) values for all three indicators in the ROC analysis indicate limited predictive ability, suggesting that oxidative balance alone may not fully account for CKD risk.

## 5. Conclusion

This study found that elevated oxidative balance indicators, specifically OBS and PAB, are associated with a reduced risk of CKD, while TAC showed no significant link. Among the indicators, OBS demonstrated the strongest predictive ability for CKD risk. These findings highlight the potential role of oxidative balance in CKD prevention. Further research is warranted to validate these associations in diverse populations and to investigate the underlying mechanisms.

## Supporting information

S1 Table Classification and scoring criteria for oxidative balance score. (DOCX)

S2 Table Classification and weighting of components in pro-oxidant and antioxidant balance. (DOCX)

S1 Fig Dose–response relationship between oxidative balance indicators and kidney function. The association between OBS (a), PAB (b), and TAC (c) with impaired kidney function (eGFR<60ml/min/1.73 m^2^), and association between OBS (d), PAB (e), and TAC (f) with proteinuria (UACR ≥30mg/g) was assessed using restricted cubic spline regression, adjusting for covariates as in Model 4. The red line indicates the odds ratio, while the pink shading represents the 95% confidence interval.
(TIF)

S2 Fig Dose–response relationship between oxidative balance score and Chronic Kidney Disease in different age groups. The association between OBS with chronic kidney disease for individuals aged <60 years (a) and ≥60 years (b).
(TIF)
