# Supplementary material for: Relationship between oxidative balance indicators and Chronic Kidney Disease
Source: PLoS One. 2025 Jan 3;20(1):e0315344. doi: 10.1371/journal.pone.0315344 (PMC11698424; doi:10.1371/journal.pone.0315344)
Supplement: S1 Table — (DOCX) [file pone.0315344.s001.docx]

**Table S1** Classification and Scoring Criteria for Oxidative Balance Score

| OBS components | Property | Male | | | Female | | |
| --- | --- | --- | --- | --- | --- | --- | --- |
|  |  | 1 | 2 | 3 | 1 | 2 | 3 |
| **Dietary OBS** | | | | | | | |
| Dietary fiber (g/d) ^a^ | A | <12.40 | 12.40-20.55 | ≥20.55 | <11.10 | 11.10-17.15 | ≥17.15 |
| Carotene (RE/d) ^a^ | A | <606.4 | 606.4-2214.6 | ≥2214.6 | <663.2 | 663.2-2434.2 | ≥2434.2 |
| Riboflavin (mg/d) ^a^ | A | <1.53 | 1.53-2.24 | ≥2.24 | <1.33 | 1.33-1.78 | ≥1.78 |
| Niacin (mg/d) ^a^ | A | <23.26 | 23.26-32.34 | ≥32.34 | <15.24 | 15.24-23.42 | ≥23.42 |
| Total folate (mcg/d) ^a^ | A | <314.5 | 314.5-498.0 | ≥498.0 | <264.5 | 264.5-374.5 | ≥374.5 |
| Calcium (mg/d) ^a^ | A | <713.5 | 713.5-1034.5 | ≥1034.5 | <623.5 | 623.5-924.0 | ≥924.0 |
| Zinc (mg/d) ^a^ | A | <9.23 | 9.23-13.48 | ≥13.48 | <6.97 | 6.97-9.97 | ≥9.97 |
| Magnesium (mg/d) ^a^ | A | <236.7 | 236.7-363.3 | ≥363.3 | <215.3 | 215.3-298.2 | ≥298.2 |
| Copper (mg/d) ^a^ | A | <0.93 | 0.93-1.39 | ≥1.39 | <0.77 | 0.77-1.21 | ≥1.21 |
| Selenium (mcg/d) ^a^ | A | <103.2 | 103.2-135.3 | ≥135.3 | <77.2 | 77.2-103.3 | ≥103.3 |
| Iron (mg/d) ^a^ | p | ≥18.26 | 11.92-18.26 | <11.92 | ≥13.25 | 9.24-13.25 | <9.24 |
| Total fat (g/d) ^a^ | P | ≥99.13 | 68.92-99.13 | <68.92 | ≥72.37 | 52.61-72.37 | <52.61 |
| Vitamin B6 (mg/d) ^a^ | A | <1.63 | 1.63-2.51 | ≥2.51 | <1.32 | 1.32-1.94 | ≥1.94 |
| Vitamin B12 (mcg/d) ^a^ | A | <3.57 | 3.57-6.82 | ≥6.82 | <2.42 | 2.42-4.86 | ≥4.86 |
| Vitamin C (mg/d) ^a^ | A | <37.24 | 37.24-93.53 | ≥93.53 | <37.57 | 37.57-90.28 | ≥90.28 |
| Vitamin E (ATE) (mg/d) ^a^ | A | <5.89 | 5.89-9.67 | ≥9.67 | <5.24 | 5.24-7.98 | ≥7.98 |
| **Lifestyle OBS** | | | | | | | |
| Physical activity (MET-minute/week) | A | <715 | 715-4035 | ≥4035 | <220 | 220-1750 | ≥1750 |
| Body mass index (kg/m2) | P | ≥30.24 | 25.93-30.24 | <25.93 | ≥31.63 | 25.29-31.63 | <25.29 |
| Alcohol | P | ≥2 drinks/d | < 2 drinks/d | <12 drinks/year | ≥1 drinks/d | < 1 drinks/d | <12 drinks/year |
| Cotinine (ng/mL) | P | ≥0.93 | 0.03-0.93 | <0.03 | ≥0.09 | 0.01-0.09 | <0.01 |

OBS: oxidative balance score; A: antioxidant; P: prooxidant; RE: retinol equivalent; ATE: alpha-tocopherol equivalent; MET: metabolic equivalent.

^a^Total intake= dietary plus supplement intakes; inclusion of supplemental intake based on the availability of supplemental intake information.
