# Supplementary material for: Relationship between oxidative balance indicators and Chronic Kidney Disease
Source: PLoS One. 2025 Jan 3;20(1):e0315344. doi: 10.1371/journal.pone.0315344 (PMC11698424; doi:10.1371/journal.pone.0315344)
Supplement: S2 Table — (DOCX) [file pone.0315344.s002.docx]

**Table S2** Classification and Weighting of Components in Pro-Oxidant and Antioxidant Balance.

| Component | Property | Categories | Weights |
| --- | --- | --- | --- |
| Smoking | P | Never | 0 |
|  |  | Used to | -1 |
|  |  | Current | -2 |
| Drinking | P | <12 drinks/year | 0 |
|  |  | <2 drinks/d for male or <1 drinks/d for female | -1 |
|  |  | ≥2 drinks/d for male or ≥1 drinks/d for female | -2 |
| BMI (kg/m^2^) | P | BMI <25 | 0 |
|  |  | 25 ≤ BMI<30 | -1 |
|  |  | BMI ≥30 | -2 |
| Fruit intake | A | Fruit intake<100g | 0 |
|  |  | 100g ≤ Fruit intake<150g | 1 |
|  |  | Fruit intake≥ 150g | 2 |
| Vegetable intake | A | Vegetable intake<150g | 0 |
|  |  | 150g ≤ Vegetable intake<250g | 1 |
|  |  | Vegetable intake≥ 250g | 2 |
| Diet quality | A | HEI2020<50 | 0 |
|  |  | 50 ≤ HEI2020 <80 | 1 |
|  |  | HEI2020 ≥ 80 | 2 |
| Physical activity (MET-min/week) | A | MET <600 | 0 |
|  |  | 600 ≤ MET<1200 | 1 |
|  |  | MET ≥ 1200 | 2 |

A: antioxidant; P: pro-oxidant;
