# Supplementary figures and images for: Relationship between oxidative balance indicators and Chronic Kidney Disease
Source: PLoS One. 2025 Jan 3;20(1):e0315344. doi: 10.1371/journal.pone.0315344 (PMC11698424; doi:10.1371/journal.pone.0315344)

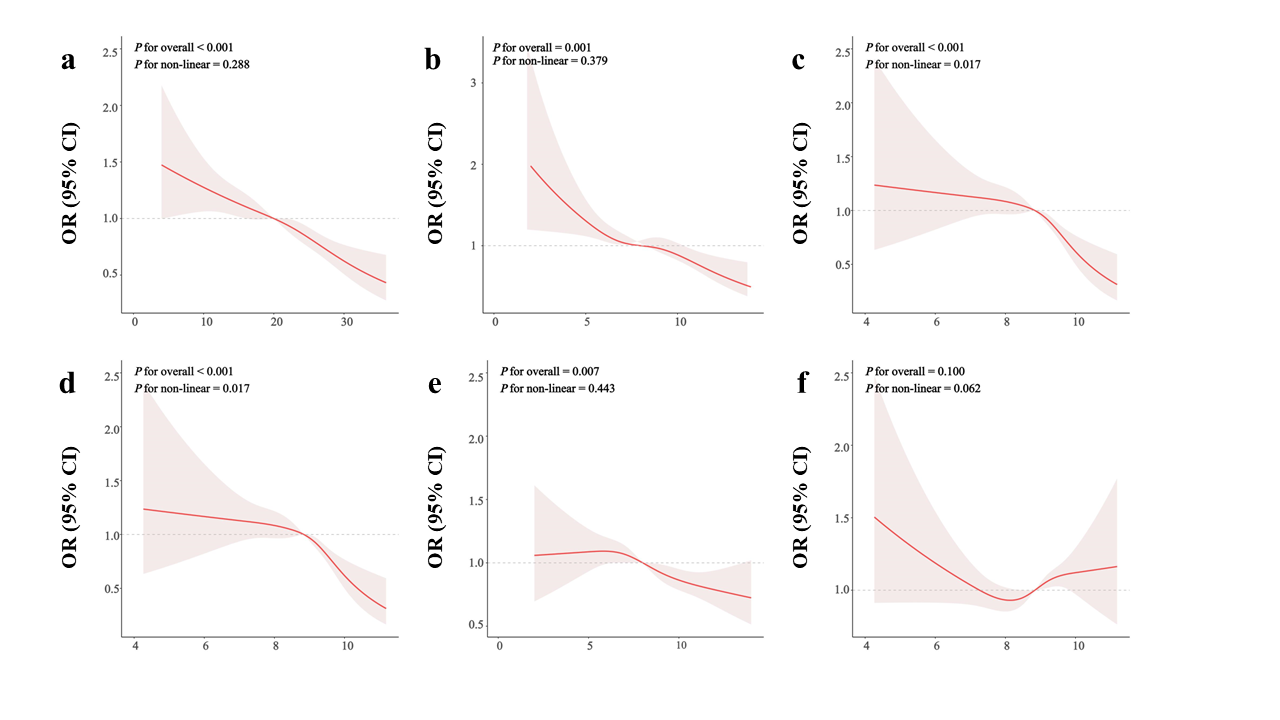

Supplement: S1 Fig — The association between OBS (a), PAB (b), and TAC (c) with impaired kidney function (eGFR<60ml/min/1.73 m2), and association between OBS (d), PAB (e), and TAC (f) with proteinuria (UACR ≥30mg/g) was assessed using restricted cubic spline regression, adjusting for covariates as in Model 4. The red line indicates the odds ratio, while the pink shading represents the 95% confidence interval. (TIF) [file pone.0315344.s003.tif]

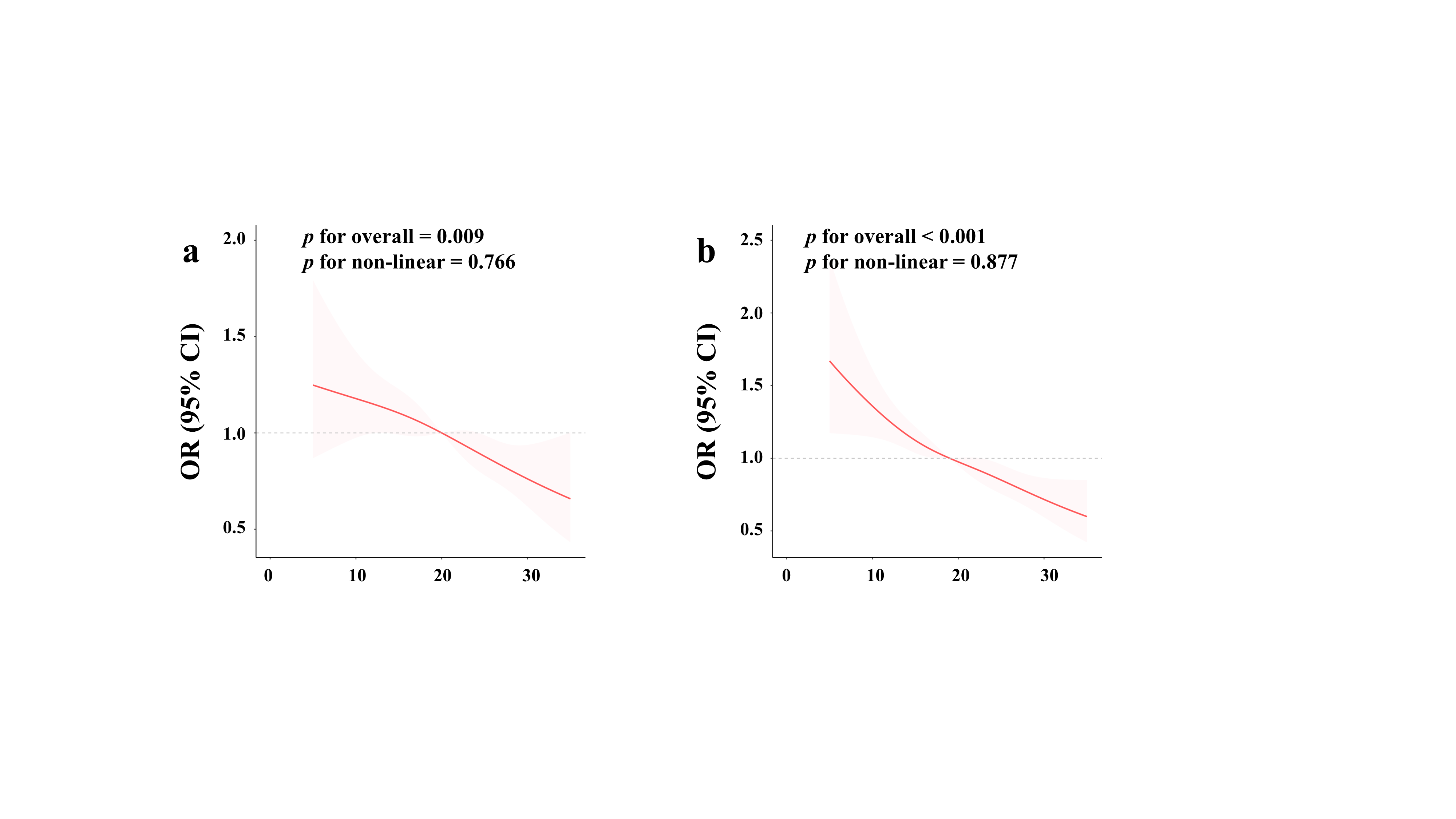

Supplement: S2 Fig — The association between OBS with chronic kidney disease for individuals aged <60 years (a) and ≥60 years (b). (TIF) [file pone.0315344.s004.tif]
